# Changes in social capital and depressive states of middle-aged adults in Japan

**DOI:** 10.1371/journal.pone.0189112

**Published:** 2017-12-07

**Authors:** Shin Nakamine, Hirokazu Tachikawa, Miyuki Aiba, Sho Takahashi, Haruko Noguchi, Hideto Takahashi, Nanako Tamiya

**Affiliations:** 1 Faculty of Human Sciences, University of Tsukuba, Tsukuba, Ibaraki, Japan; 2 JSPS Research Fellow, Chiyoda-ku, Tokyo, Japan; 3 Department of Psychiatry, University of Tsukuba, Tsukuba, Ibaraki, Japan; 4 Faculty of Human Sciences, Toyo Gakuen University, Bunkyo-ku, Tokyo, Japan; 5 School of Political Science and Economics, Waseda University, Shinjuku-ku, Tokyo, Japan; 6 Natinal Institute of Public Health, Wako, Saitama, Japan; 7 Faculty of Medicine, University of Tsukuba, Tsukuba, Ibaraki, Japan; Universita degli Studi di Pisa, ITALY

## Abstract

The present study examines the relationships between changes in bonding and bridging types of social capital and depressive states among middle-aged adults in Japan using a nationally representative sample. Data was collected from a nationwide, population-based survey conducted from 2005 to 2013 in nine annual waves. A total of 16,737 middle-aged men and 17,768 middle-aged women provided data. They reported about depressive states, measured by Kessler 6 scores, and bonding and bridging types of social capital, measured by reported participation in different social activities. Latent growth modeling was conducted to examine relations between changes in bonding and bridging types of social capital and depressive states within individuals across the nine waves. The results showed that, for both men and women, increases in bonding social capital were associated with decreases in depressive states, while changes in bridging social capital were not related to changes in depressive states. In addition, the results showed that changes in bonding social capital, but not bonding social capital at the baseline, affected changes in depressive states. Future studies should take changes in social capital as an independent variable into consideration.

## Introduction

Depression is one of the most prevalent of all psychiatric disorders [1]. Depression was ranked as the single most burdensome disease in the world in terms of disability-adjusted life years (DALY) among middle-aged adults [2]. It adversely affected not only physical health [3] but also the quality of interpersonal relationships [4, 5]. Thus, depression significantly influences individual well-being.

Depression in older adults is different from depression earlier in the lifespan in that the former is more closely associated with risk factors such as suicide, which are in turn related to increased mortality [6]. Among older adults, the prevalence rate of depression in middle-aged adults was higher than in the elderly [7, 8]. In addition, the prevalence rate scarcely declined with age [7]. Therefore, it is important to examine the factors that prevent and treat depression in middle-aged adults.

In Japan, the prevalence rate of mental disorders has been increasing recently [9]. The total number of people with mood disorders was estimated as 1,116,000 in 2014, of whom 274,000 (about 25%) were middle-aged adults aged 50 to 64 [10]. Preventing depression is important to enhance the quality of life not only among the current middle-aged cohort, but also the future generation aged 65 and older, which will compose more than 30% of the entire Japanese population in the next several decades [11].

Concern about social capital as an important factor for preventing depression has been growing over the past few decades. Social capital refers to social networks, and the trust and norms derived from them [12]. Many research studies have shown that greater social capital at the individual level is associated with less depression [13, 14], although social capital is often treated as a collective-level concept [12].

According to Oshio, prior studies examining the association between individual-level social capital and health can be divided into cross-sectional studies and prospective cohort studies [15]. Cross-sectional studies address the contemporaneous association between social capital and health [16, 17]. In contrast, prospective cohort studies focus on how social capital in the baseline year explains health outcomes or changes in the follow-up years [14, 18]. Murayama et al. have pointed out that longitudinal studies are required to understand the causal effect of social capital on health [18].

Most longitudinal studies addressing the association between individual-level social capital and depression have also examined the causal effect of social capital in the baseline year on depression or changes in the follow-up years, similar to Oshio [15]. However, individual-level social capital, as well as depression, change over time [19, 20]. Nevertheless, there has been few studies examining the association between changes in both individual-level social capital and depression.

This study examined the association between changes in individual-level social capital and depression among middle-aged adults, using nationally representative data in Japan. Especially, we focused on individual-level bonding and bridging aspects of social capital. Bonding social capital is derived from relationships among homogenous people who are similar in terms of sociodemographic or social characteristics (e.g., age, ethnicity, and social class), whereas bridging social capital is derived from relationships among heterogeneous people who do not necessarily share similar identities [12, 21]. Previous studies have demonstrated that bonding social capital negatively affected depression and bridging social capital was not associated with depression in Japan [15, 18]. Based on these results, it is likely that increases in bonding social capital over time are associated with decreases in depression, while changes in bridging social capital are not related to depression.

## Method

### Study population and procedure

This study used nine-wave panel data obtained from a nationwide, population-based survey, the “Longitudinal Survey of Middle-aged and Elderly Persons (LSMEP)”, which has been conducted since 2005 by the Japanese Ministry of Health, Labour and Welfare (MHLW) in Japan. Respondents to the survey were extracted randomly through a stratified two-stage sampling. First, 2,515 districts in 2005 were selected at random from the entire 5,280 districts surveyed by the population-based “Comprehensive Survey of the Living Conditions of People on Health and Welfare” conducted by the MHLW in 2004. Second, 40,877 residents were chosen randomly from those aged 50 to 59 living in each selected district in proportion to the population size.

In 2005 the questionnaires were dropped off at the respondents’ homes by enumerators. Then, the enumerators collected the self-completed questionnaire several days later from 34,240 (response rate: 83.8%). As of 2006, the method had changed from a drop-off to a mail survey and the questionnaires were mailed only to those who had responded to the first survey in 2005. No new respondents were added after the first year of the survey, and response rate at the latest year of 2013 declined to 58.0%.

This research project received official approval to use secondary data from the Statistics and Information Department of the MHLW under Tohatsu-1218-1 on December 18, 2015. An ethical review of the LSMEP is not required according to the Ethical Guidelines for Epidemiological Research of the Japanese government [22]. In addition, this study was approved by the official ethical review board of the University of Tsukuba (*I No Rinri Iinkai*, Document No. 1009).

### Measures

Please see S1 Appendix for more detailed information about the survey question used in the study.

#### Depressive state

Depressive state was assessed using the Japanese version of the Kessler 6 (K6) scale [23]. Respondents were asked to rate six items on a five-point scale (0 = *none of the time* to 4 = *all of the time*): During the past 30 days, about how often did you feel (1) nervous, (2) hopeless, (3) restless or fidgety, (4) so depressed that nothing could cheer you up, (5) that everything was an effort, and (6) worthless? Then, the sum of the reported scores (range: 0–24) was calculated and defined as the K6 score. Higher K6 scores reflect higher levels of depressive state. The Cronbach’s alpha coefficient throughout the nine waves was α > .88 for both men and women.

#### Social capital

To construct social capital measures, this study used questions about participation in social activities, which had been used in previous studies in Japan [15, 24, 25]. Respondents were asked whether they participated in each of six types of social activities within the past one year from the date of the survey: (1) hobby or entertainment, (2) sports or physical exercises, (3) community activities, (4) childcare support, or educational or cultural activities, (5) support for the elderly, and (6) others. Multiple answers were permitted. If respondents reported ‘yes’, they were asked to indicate with whom, if anyone, they participated in each activity by choosing (A) alone, (B) family members or friends, (C) workplace colleagues, (D) in a neighborhood association, or (E) in a non-profit organization or public-service corporation. Again, multiple answers were permitted. The number of social activities they participated with each of (B)-(E) was calculated (ranges: 0–6). According to a preceding study [26], the difference between bonding and bridging social capital can be defined as the distinction of a geographical character: Bonding implies *within* community relations and bridging implies *extra* (outside) community relations. However, another preceding study [27] defined bonding and bridging social capital as the distinction between background characters as well as Oshio [15] and Szreter and Woolcock [21]: Bonding social capital can be defined as relationship within homogenous group such as family members, neighbors, and close friends and colleagues, whereas bridging social capital can be defined as linking people with different ethnic and occupational backgrounds. In addition, the former study [26] implied that it was possible to define bonding and bridging social capital as shared or unshared cultural backgrounds, although the study defined bonding and bridging as the distinction of a geographical character. Following preceding studies [15, 27], the number of social activities they participated with family members or friends, workplace colleagues, or in a neighborhood association was considered equivalent to bonding social capital, whereas the number of social activities they participated in a non-profit organization or a public-service corporation was considered equivalent to bridging social capital.

#### Demographic and socioeconomic status and chronic disease diagnosis

Demographic and socioeconomic status included gender, age (calculated from the month and year of birth), marital status (1 = married, 0 = unmarried), educational achievement (1 = college graduate or above, 0 = high school graduate or below), working hours of a week, and house ownership (1 = yes, 0 = no). These variables measured at Wave 1 were used.

The survey asked respondents whether they suffered from each of the following diagnoses: diabetes, heart disease, stroke, hypertension, hyperlipidemia, and cancer. A binary variable for chronic disease diagnosis was constructed by allocating 1 to respondents who reported at least one of the diagnoses and 0 to otherwise. These variables measured at Wave 1 were used.

### Statistical analysis

All statistical analyses were conducted separately according to gender because depression was not likely to decline with age in women compared with men [7] and social capital had a more significant effect on depression in women compared to men [25]. Descriptive analyses were performed using SPSS 21.0 for Windows. For the descriptive statistics and the construction of depressive status and social capital variables, we used pairwise deletion of missing data.

To examine changes in depressive states and social capital across the nine waves, Latent Growth Modeling (LGM) with Mplus version 7.4 was used, based on full information maximum-likelihood parameter estimation to handle missing data. First, we compared a linear growth model and a quadratic growth model of depressive state and social capital with LGM. We used Comparative Fixed Index (CFI), Root Mean Square Error of Approximation (RMSEA), and Akaike’s Information Criterion (AIC) to evaluate the goodness of fit for the tested models. CFI assumes that all latent variables are uncorrelated (null/ independence model) and compares the sample covariance matrix with this null model. The CFI ranges between 0.0 and 1.0 with values closer to 1.0 indicating a good fit. A value of CFI ≥ 0.95 is recommended as indicative of a good fit [28]. RMSEA indicates how well the model would fit the population’s covariance matrix. RMSEA favors parsimony in that it will choose the model with the lesser number of parameters [28]. A value of RMSEA ≤ 0.07 is recommended [28]. AIC is used to compare plural models, which comprise the same variables. The model which has a smaller value for AIC has a better fit than the other models.

**Fig 1:**
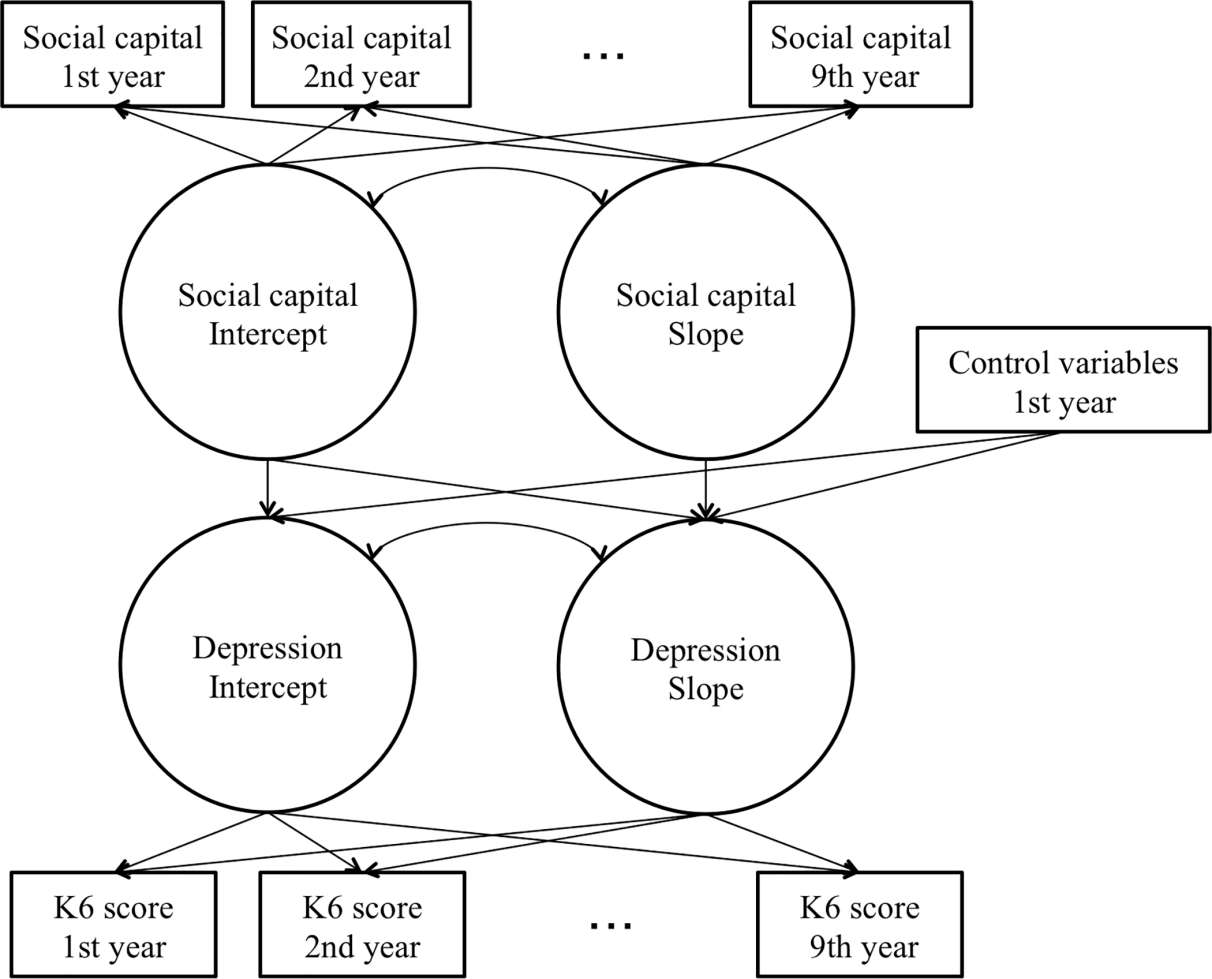
Outline of the tested model using latent growth modeling.

To examine relations between changes in depressive state and social capital, we regressed the latent variables of depressive state that reflected the baseline (intercept) and the growth factor (slope) on age, marital status, educational achievement, working hours, house ownership, chronic disease diagnosis, and the latent variable of social capital that reflected the baseline. In addition, we regressed the growth factor of depressive state on the growth factor of social capital (Fig 1).

## Results

Table 1 summarizes the demographic and socioeconomic status and chronic disease diagnosis measures.

**Table 1:** Descriptive statistics on demographic status, socioeconomic status, and the diagnosis of chronic disease by gender.

	Men (*n* = 16737)			Women (*n* = 17768)		
	*M* (*SD*)	*n* (%)	Missing data	*M* (*SD*)	*n* (%)	Missing data
Age (years)	54.83 (2.74)	-	0	54.55 (2.75)	-	0
Marital status (married)	-	14240 (85.1)	352	-	14690 (82.7)	403
Educational achievement (college or above)	-	3647 (21.8)	1389	-	1388 (7.8)	1436
Working hours	43.25 (16.82)	-	808	23.08 (20.28)	-	937
House ownership (yes)	-	13768 (82.3)	417	-	14769 (83.1)	465
Chronic disease diagnosis (yes)	-	5494 (32.8)	321	-	4712 (26.5)	369

### Changes in depressive state and social capital

Descriptive statistics of depressive state and social capital across the nine waves are shown in Table 2.

**Table 2:** Descriptive statistics about depressive state across the nine waves by gender.

		Wave 1	Wave 2	Wave 3	Wave 4	Wave 5	Wave 6	Wave 7	Wave 8	Wave 9
Men (*n* = 16737)										
Depressive state (K6)	*n*	15272	14264	14066	13616	13189	11985	11535	10905	10711
	*M*	2.93	3.05	3.18	3.16	3.11	3.05	3.03	2.95	2.84
	*SD*	3.97	4.06	4.09	4.04	4.04	4.04	3.93	3.94	3.88
Social activities participating with	*n*	14966	14133	13566	13043	12606	11699	11257	10602	10515
family members or friends	*M*	0.50	0.54	0.57	0.57	0.60	0.74	0.75	0.78	0.77
	*SD*	0.72	0.74	0.75	0.75	0.77	0.89	0.89	0.91	0.92
workplace colleagues	*M*	0.16	0.16	0.15	0.15	0.13	0.21	0.20	0.19	0.18
	*SD*	0.44	0.44	0.43	0.42	0.40	0.51	0.51	0.50	0.49
members of a neighborhood association	*M*	0.31	0.34	0.36	0.37	0.38	0.59	0.60	0.62	0.63
	*SD*	0.61	0.63	0.65	0.67	0.67	0.89	0.90	0.92	0.95
members of a non-profit organization or public-service corporation	*M*	0.05	0.05	0.05	0.05	0.06	0.10	0.11	0.12	0.11
	*SD*	0.26	0.28	0.27	0.28	0.28	0.41	0.42	0.45	0.44
Women (*n* = 17768)										
Depressive state (K6)	*n*	16233	14972	15122	14588	14302	13384	12930	12409	12221
	*M*	3.24	3.48	3.60	3.56	3.55	3.56	3.68	3.55	3.47
	*SD*	4.03	4.23	4.21	4.15	4.18	4.24	4.23	4.21	4.13
Social activities participating with	*n*	15863	14700	14301	13873	13461	12842	12479	11942	11787
family members or friends	*M*	0.67	0.75	0.76	0.77	0.79	0.96	0.95	0.97	0.99
	*SD*	0.80	0.81	0.82	0.83	0.83	0.96	0.96	0.98	1.02
workplace colleagues	*M*	0.08	0.08	0.08	0.07	0.07	0.12	0.11	0.11	0.10
	*SD*	0.32	0.31	0.30	0.29	0.28	0.40	0.38	0.38	0.38
members of a neighborhood association	*M*	0.28	0.30	0.32	0.33	0.33	0.53	0.52	0.55	0.56
	*SD*	0.57	0.59	0.60	0.62	0.62	0.85	0.82	0.86	0.90
members of a non-profit organization or public-service corporation	*M*	0.06	0.07	0.06	0.06	0.07	0.11	0.12	0.12	0.12
	*SD*	0.29	0.31	0.30	0.29	0.30	0.44	0.44	0.46	0.49

To examine changes in depressive state across the nine waves, LGMs were conducted by gender (Table 3). For men, the linear growth model had a fair to good fit, CFI = .979, RMSEA = .029, AIC = 583313.83. The intercept and linear slope means were 3.09 and 0.01. The quadratic growth model also had a fair to good fit, CFI = .994, RMSEA = .016, AIC = 582526.86. The intercept, linear slope and quadratic slope means were 2.96, 0.12, and -0.01. For women, the linear growth model had a fair to good fit, CFI = .976, RMSEA = .034, AIC = 639711.75. The intercept and linear slope means were 3.42 and 0.04. The quadratic growth model also had a fair to good fit, CFI = .992, RMSEA = .021, AIC = 638693.30. The intercept, linear slope and quadratic slope means were 3.28, 0.16, and -0.02. Since the quadratic slope means were quite small for men and women, the linear growth models were adopted because they were more straightforward to interpret than the quadratic growth models.

**Table 3:** Estimated values of the linear growth model (linear) and the quadratic growth model (quadratic) for depressive state with latent growth modeling by gender.

	Men				Women			
	Linear		Quadratic		Linear		Quadratic	
Means								
Intercept	3.087	***	2.964	***	3.416	***	3.281	***
Linear slope	0.014	**	0.124	***	0.044	***	0.163	***
Quadratic slope			-0.014	***			-0.015	***
Variance								
Intercept	10.063	***	9.738	***	11.143	***	11.073	***
Linear slope	0.099	***	0.600	***	0.107	***	0.709	***
Quadratic slope			0.008	***			0.009	***
Correlation								
Intercept with linear slope	-.227	***	-.132	***	-.201	***	-.168	***
Intercept with quadratic slope			.024				.075	**
Linear slope with quadratic slope			-.909	***			-.916	***
Model fit								
CFI	.979		.994		.976		.992	
RMSEA	.029		.016		.034		.021	
AIC	583313.83		582526.86		639711.75		638693.30	

**p < .01

***p < .001.

To examine changes in social capital across the nine waves, LGMs were conducted by gender (Table 4). For men, the linear growth model had a fair to good fit, CFI = .955, RMSEA = .018, AIC = 580609.82. The quadratic growth model also had a fair to good fit, CFI = .967, RMSEA = .016, AIC = 578743.43. For women, the linear growth model had a fair to good fit, CFI = .950, RMSEA = .018, AIC = 598958.43. The quadratic growth model also had a fair to good fit, CFI = .965, RMSEA = .015, AIC = 596735.30. Although the quadratic growth models had a slightly better goodness of fit than the linear growth models for both men and women, the quadratic slope means were quite small. Thus, the linear growth models were adopted because the models were more straightforward to interpret than the quadratic growth models.

**Table 4:** Estimated values of the linear growth model (linear) and the quadratic growth model (quadratic) for social capital by gender.

	**Men**															
	**Linear**								**Quadratic**							
	**Bonding 1**		**Bonding 2**		**Bonding 3**		**Bonding 4**		**Bonding 1**		**Bonding 2**		**Bonding 3**		**Bonding 4**	
Means																
Intercept	0.482	***	0.145	***	0.279	***	0.040	***	0.484	***	0.152	***	0.297	***	0.047	***
Linear slope	0.034	**	0.004	***	0.039	**	0.007	***	0.031	***	-0.003	*	0.020	***	0.000	
Quadratic slope									0.000		0.001	***	0.003	***	0.001	***
Variance																
Intercept	0.233	***	0.080	***	0.187	***	0.027	***	0.228	***	0.078	***	0.193	***	0.032	***
Linear slope	0.004	***	0.002	***	0.006	***	0.001	***	0.018	***	0.005	***	0.017	***	0.004	***
Quadratic slope									0.000	***	0.000	***	0.000	***	0.000	***
Correlation																
Intercept with linear slope	-.033		-.326	***	.022		-.082		-.079		-.242	***	-.116	**	-.332	***
Intercept with quadratic slope									.076	*	.085		.161	***	.330	***
Linear slope with quadratic slope									-.868	***	-.820	***	-.820	***	-.830	***
Model fit																
CFI	.955								.967							
RMSEA	.018								.016							
AIC	580609.82								578743.43							
	**Women**															
	**Linear**								**Quadratic**							
	**Bonding 1**		**Bonding 2**		**Bonding 3**		**Bonding 4**		**Bonding 1**		**Bonding 2**		**Bonding 3**		**Bonding 4**	
Means																
Intercept	0.663	***	0.070	***	0.247	***	0.050	***	0.663	***	0.076	***	0.269	***	0.059	***
Linear slope	0.036	***	0.004	***	0.035	***	0.007	***	0.036	***	-0.002		0.012	***	-0.001	
Quadratic slope									0.000		0.001	***	0.003	***	0.001	***
Variance																
Intercept	0.306	***	0.031	***	0.139	***	0.032	***	0.312	***	0.034	***	0.160	***	0.038	***
Linear slope	0.005	***	0.001	***	0.005	***	0.001	***	0.023	***	0.003	***	0.020	***	0.004	***
Quadratic slope									0.000	***	0.000	***	0.000	***	0.000	***
Correlation																
Intercept with linear slope	-.087	***	-.259	***	-.006		-.133	**	-.166	***	-.369	***	-.292	***	-.373	***
Intercept with quadratic slope									.142	***	.274	***	.313	***	.337	***
Linear slope with quadratic slope									-.870	***	-.876	***	-.861	***	-.820	***
Model fit																
CFI	.950								.965							
RMSEA	.018								.015							
AIC	598958.43								596735.30							

Bonding 1: the number of social activities participating with family members or friends. Bonding 2: the number of social activities participating with workplace colleagues. Bonding 3: the number of social activities participating in a neighborhood association. Bridging: the number of social activities participating in a non-profit organization or public-service corporation.

*p < .05

**p < .01

***p < .001.

### Relationship between changes in depressive state and social capital across the nine waves

To examine the relationship between changes in depressive state and social capital, LGMs were conducted separately for men and women (Table 5). We regressed the intercept and linear slope of depressive state on age, marital status, educational achievement, job status, house ownership, chronic disease diagnosis, and the intercept of social capital. In addition, we regressed the linear slope of depressive state on the linear slope of social capital.

**Table 5:** Estimated associations between social capital and depressive state.

	K6 (depressive state)											
	Men						Women					
	Intercept			Slope			Intercept			Slope		
	*b*	*SE*		*b*	*SE*		*b*	*SE*		*b*	*SE*	
Bonding1												
Intercept	-0.879	0.067	***	0.013	0.010		-1.011	0.063	***	0.003	0.009	
Slope				-0.801	0.102	***				-0.823	0.086	***
Bonding 2												
Intercept	-0.716	0.115	***	0.033	0.017		-0.548	0.214	*	0.027	0.029	
Slope				-0.571	0.138	***				-0.427	0.216	*
Bonding 3												
Intercept	-0.217	0.071	**	0.025	0.010	*	-0.188	0.093	*	0.034	0.013	*
Slope				-0.309	0.071	***				-0.228	0.078	*
Bridging												
Intercept	0.169	0.215		0.050	0.031		-0.362	0.188		0.029	0.029	
Slope				-0.064	0.157					-0.148	0.154	
Control variables												
Age	-0.101	0.011	***	0.000	0.002		-0.092	0.011	***	-0.003	0.002	*
Married	-0.590	0.104	***	0.025	0.014		-0.441	0.094	***	0.023	0.012	
Education	0.025	0.069		-0.037	0.009	***	0.170	0.103		-0.006	0.015	
Working hours	-0.015	0.002	***	0.000	0.000		-0.009	0.002	***	0.000	0.000	
House ownership	-0.351	0.090	***	-0.019	0.013		-0.270	0.098	**	0.012	0.013	
Diagnosis	0.607	0.063	***	-0.013	0.009		0.668	0.070	***	-0.009	0.009	
Model fit												
CFI	.954						.950					
RMSEA	.019						.019					

Bonding 1: the number of social activities participating with family members or friends. Bonding 2: the number of social activities participating with workplace colleagues. Bonding 3: the number of social activities participating in a neighborhood association. Bridging: the number of social activities participating in a non-profit organization or public-service corporation.

*p < .05

**p < .01

***p < .001.

For men, the intercepts of bonding social capital (bonding 1, bonding 2, and bonding 3) were negatively related to the intercepts of depressive state, *b*s = -0.844, -0.596, and -0.203, *p*s < .01. The intercept of bridging social capital was not related to the intercept of depressive state, *b* = 0.147, *p* = .487. This indicates that depressive state was lower at the baseline while bonding social capital was higher at the baseline. The results also showed that the slopes of bonding social capital negatively affected the slope of depressive state, while the intercept and slope of bridging social capital did not affect the slope of depressive state. In addition, the intercept of bonding social capital (bonding 3: the number of social activities participating in a neighborhood association) positively affected the slope of depressive state. Thus increases in bonding social capital were associated with decreases in depressive state, while depressive state increased when bonding social capital was higher at the baseline.

For women, the same results were found. In other words, depressive state was lower at the baseline when bonding social capital was higher at the baseline. In addition, increases in bonding social capital were associated with decreases in depressive state, while depressive state increased when bonding social capital was higher at the baseline.

## Discussion

The purpose of this study was to examine the association between changes in bonding and bridging types of social capital and depressive states among middle-aged adults in Japan using a nine-wave nationally representative data. Unlike most previous studies, we focused on changes in social capital and confirmed that changes in social capital, especially bonding social capital, are important for preventing depressive states.

Regarding changes in social capital and depressive states across the nine waves, linear growth models were adopted, regardless of gender. The results indicated that bonding and bridging types of social capital and depressive states slightly increased on average for both men and women as time went on.

For associations between bonding social capital and depressive state at the baseline, which corresponded to the contemporaneous association between bonding social capital and depression, we confirmed that bonding types of social capital at the baseline (intercepts) were negatively associated with depressive state at the baseline for both men and women. These results support previous findings [15, 16, 29]. Bonding social capital refers to aspects of “inward-looking” social networks that reinforce homogeneous groups [21]. As described in detail in previous study [18], the similarity among local residents is of greater benefit than dissimilarity, because shared personal characteristics elicit perceptions of trust and social resemblance that might foster the development of a social support system in the community [30]. It is known that social support buffers against stress [31] and therefore, it was expected that bonding social capital would be negatively related to depression.

On the other hand, bridging social capital at the baseline was not associated with depressive state at the baseline for either men or women, as has been described in previous studies [16, 29]. Bridging social capital refers to “outward-looking” social networks with different members that do not necessarily share similar identities [21]. Bridging social capital is considered important for gathering diverse information and obtaining outside assistance for addressing significant challenges [32], whereas dissimilarities in bridging tie may impede improvements in health outcomes by hindering the development of social support and mutual respect [16, 30]. Consequently, it is likely that there was no association between bridging social capital and depressive state at the baseline.

For associations between changes in social capital and depressive state, changes in bridging social capital were not related to changes in depressive state, while changes in bonding social capital were negatively related to changes in depressive state for both men and women. Only increases in bonding social capital were related to a decrease in depressive state within individuals. As mentioned above, bonding social capital may foster the development of a social support system in a community [18, 30], which buffers stress [31]. It is likely that an increase in bonding social capital leads to the development of a social support system, which leads to a decrease in the depressive state. On the other hand, bridging social capital refers to “outward-looking” social networks with different members that do not necessarily share similar identities [21]. Such differences could be important for gathering diverse information and obtaining outside assistance for addressing significant challenges [32], which might positively affect mental health. On the other hand, dissimilarities among people in heterogeneous groups might impede improvements in health outcomes by hindering the development of social support and mutual respect [16, 30]. Also, dissimilarities could lead to interpersonal conflicts if individuals cannot accept a diversity of social characteristics. Demerits such as these might also negatively affect mental health. Therefore, the positive effects of bridging social capital on mental health might be offset by its negative effects, and therefore, it is possible that changes in bridging social capital were not associated with changes in the depressive state.

Bridging social capital at the baseline did not affect changes in depressive state among men and women. This result supports the previous findings [12, 15]. However, bonding social capital at the baseline mostly did not affect changes in depressive state among men and women, which does not support the previous findings [15, 18]. This result suggests that it is necessary to focus on not only change in depressive state but also change in social capital. Since most longitudinal studies examined the effect of social capital in the baseline year on depression or change in follow-up years [15], it is not clear whether individuals with higher bonding social capital in the baseline year become less depressive in the follow-up years or whether there is an increase in bonding social capital within individuals which affected a lower depressive state. Our results support the latter. In other words, it suggests that advancing or maintaining bonding social capital is important for preventing depression.

However, an element of bonding social capital at the baseline, which was the number of social activities that they participated in a neighborhood association, slightly affected the increase in depressive state among men and women. According to previous studies, the effect of social capital on depression varies depending on the types of social capital [13, 33]. In Japan, a neighborhood association is an institutionalized organization because there are institutionalized agreements, a specified structure and enterprises, as well as rules about elections and an account keeping [34, 35]. On the other hand, institutionalized agreements and rules do not exist in relationships such as those between family, friends, and workplace colleagues. Therefore, neighborhood associations are more formal relationships than family, friends, and workplace colleagues. Such formal relationships often impose obligations on members to obey group norms, and such obligations often prevent people from expressing their opinions [36, 37]. Moreove people that do not fulfill obligations sometimes experience social exclusion [38]. Therefore, obligations such as these might have a negative influence on mental health [39, 40]. As a result, the number of social activities in a neighborhood association might have affected the increase in the depressive state.

In summary, the results showed that increases in bonding social capital were related to a decrease in depressive state within an individual, while a change in bridging social capital was not related to changes in depressive state. However, the results also showed that a certain type of bonding social capital at the baseline affected an increase in depressive state. This study extended knowledge regarding the effect of social capital on depression by indicating that it was important to focus not only on change in depression but also on change in social capital. Furthermore, the present study clarified the dark side of bonding social capital by showing the effect of one type of social capital that increased depression.

This study has some limitations. First, it is not clear whether depressive state decreases because of increases in social capital or social capital increases because of a decrease in depressive state. So, future studies are needed to examine the direction of this causality. Second, the K6 (depressive state) mean was very low. According to Kessler et al., the cut-point on the K6 is 13 or more to detect serious mental illness [41]. Thus, the participants in this study might be psychologically distressed, not depressive state. However, Kawakami et al. indicated that the optimal cut-point was 5 or more for screening for depression to prevent suicide [42]. Thus, although our findings are important for preventing some depression, more evidence is needed to judge whether the results in our study can be applied to middle-aged adults with serious depressive disorders. Third, there are several components of the multidimensional concept of social capital that were not captured in this study (e.g., trust, sense of belonging). Although our study constructed social capital measures following the example of previous studies [15, 24–27], it is necessary to examine the effect of social capital including more components on depression to understand the effect more exactly. Finally, factors such as retirement, which affect social capital was not examined in this study. For example, the average age of participants in our study was about 55 years at the baseline. During the nine years of the survey, there must have been some people that retired, and retirement must affect the daily life activities of retired people. Therefore, it is suggested that future studies should examine relationships between social capital and mental health while taking other factors such as retirement into consideration.

Despite these limitations, our study suggests the importance of change in social capital. It is hoped that future studies would take change in social capital as an independent variable into consideration. Moreover, an increase in bonding types of social capital would be effective to decrease a depressive state. These findings may be useful for preventing depression in middle-aged adults in Japan, which may contribute to improving their quality of life.

## Supporting information

S1 Appendix Information on survey questions used in the study in Japanese and English. (DOCX)
