# Supplementary material for: Changes in social capital and depressive states of middle-aged adults in Japan
Source: PLoS One. 2017 Dec 7;12(12):e0189112. doi: 10.1371/journal.pone.0189112 (PMC5720718; doi:10.1371/journal.pone.0189112)
Supplement: S1 Appendix — (DOCX) [file pone.0189112.s001.docx]

**Supporting information**

In regard to the survey questions used in the study in English, Please see Oshio [1] and Takeda, Noguchi, Monma, & Tamiya [2].

In regard to the survey questions in Japanese, Please see Home Page of Ministry of Health, Labour and Welfare (http://www.mhlw.go.jp/toukei/chousahyo/index.html#00450045).

**Reference**

1. Oshio T. The association between individual-level social capital and health: Cross-sectional, prospective cohort and fixed-effects models. J Epidemiol Community Health. 2016; 70: 25-30. doi: 10. 1136/jech-2015-205962
2. Takeda F, Noguchi H, Monma T, Tamiya N. How possibly do leisure and social activities impact mental health of middle-aged adults in Japan? An evidence from a national longitudinal survey. PLOS ONE. 2015; 10: e0139777. doi: 10. 1371/journal. pone. 0139777
